# Restoring Enamel Strength: A Knoop Hardness Number Evaluation of Remineralizing Toothpastes

**DOI:** 10.7759/cureus.70434

**Published:** 2024-09-29

**Authors:** Monicaa Azhagiri, Suganya Panneer Selvam, Ramya Ramadoss, Sandhya Sundar

**Affiliations:** 1 Oral Biology, Saveetha Dental College and Hospitals, Saveetha Institute of Medical and Technical Sciences, Saveetha University, Chennai, IND

**Keywords:** demineralization, enamel, fluoride, remineralization, toothpaste

## Abstract

Aim

This investigation compares and assesses the microhardness of human dental enamel after the use of two types of widely available remineralizing toothpaste.

Methodology

Thirty extracted anterior incisors were chosen as study samples and split into three groups: Control (Group 1), Enafix (EX-Group 2), and Sensodyne Repair (SR-Group 3). All of the sample groups were assessed using a Knoop indenter at baseline, after demineralization, and then again after remineralization. Then, to compare the outcomes for the variation in Knoop hardness, a one-way analysis of variance (ANOVA) was employed.

Results

The Control group exhibited the highest mean microhardness (472.00±17.783), indicating superior enamel integrity and the least variability. The EX group showed a lower mean microhardness of 340.40±40.368, demonstrating effective remineralization but with greater variability. Statistical analysis using ANOVA revealed highly significant differences between the groups, with an F-statistic of 104.292 and a p-value of 0.000, indicating that the variations in microhardness among the groups were statistically significant.

Conclusion

Both SR and EX toothpaste can effectively help dental enamel regain its hardness and remineralization following demineralization with slightly superior activity from EX.

## Introduction

Dental caries, a common oral health issue, is characterized by the erosion of tooth enamel caused by acid produced by bacterial metabolic processes. The dynamic interplay between demineralization and remineralization is pivotal in upholding enamel integrity [1]. The equilibrium between these processes determines the advancement or reversal of early carious lesions. Remineralization, the intrinsic restoration mechanism of enamel, entails the deposition of minerals, primarily calcium and phosphate, to replenish the mineral content lost during demineralization. This process is imperative for preserving the hardness and structural soundness of the enamel [2].

In recent years, there has been substantial interest in the advancement of remineralization therapies, particularly using remineralization toothpaste. These toothpaste formulations frequently encompass fluoride, calcium phosphate, and other active agents engineered to augment remineralization [3]. Fluoride has long been recognized as a crucial element in promoting enamel remineralization by fostering the formation of fluorapatite, a mineral phase that is more resistant to acid. However, the effectiveness of these formulations varies based on their composition, concentration of active ingredients, and mode of delivery [4,5].

The Knoop hardness number (KHN) is defined in the Glossary of Prosthodontic Terms as a value that quantifies a material's hardness. It is determined by measuring the length of the long diagonal of the indentation made by a rhombic-based, elongated diamond indenter. The test is performed under a specified load, making it useful for evaluating the hardness of brittle materials, thin layers, or materials where only a small indentation is permissible [6]. The KHN method involves applying a controlled load to the enamel surface using a diamond-shaped indenter, which creates an indentation. The dimensions of this indentation are measured to calculate the hardness value. By comparing KHN values before and after treatment with a remineralizing toothpaste, researchers can quantitatively determine the degree of enamel remineralization and assess the toothpaste's effectiveness in enhancing enamel hardness [7,8].

This study aims to evaluate the effectiveness of a remineralization toothpaste in restoring enamel hardness using KHN measurements. By analyzing changes in KHN, the study will provide empirical evidence regarding the toothpaste's ability to promote remineralization and protect against further enamel demineralization. Understanding the performance of these formulations is essential for developing preventive strategies against dental caries and improving overall oral health outcomes.

## Materials and methods

Ethical clearance and sample collection

The study specimens, comprising randomly selected 30 extracted central incisor teeth, were obtained from the college archives, tooth repository, Department of Oral Biology, Saveetha Dental College and Hospitals, Chennai. As this is an in vitro study, ethical approval was not required. However, the teeth were extracted after obtaining informed consent from the patient.

Preparation of the demineralizing solution

A demineralizing solution was prepared, consisting of 2.2 mM calcium chloride (CaCl₂·2H₂O), 2.2 mM monosodium phosphate (NaH₂PO₄·7H₂O), and 0.5 M acetic acid. The pH was adjusted to 4.4 using 50% potassium hydroxide (KOH). The pH of the solution was measured using a digital pH meter before and after preparation, with a phosphate buffer solution at pH 7.0 used for calibration of the instrument.

Sample preparation

Sample preparation involved sectioning the teeth using a hard tissue microtome (Leica SP 1100) and mounting the sections onto acrylic molds. At this stage, the initial KHN was recorded. The samples were then immersed in a demineralizing solution for 72 hours. Following demineralization, a toothbrush simulator was used to treat the samples with Sensodyne Repair (SR), Enafix (EX), and one group left untreated as a Control. After treatment, the KHN was recorded again to assess the effects of each treatment. 

Measurement of baseline knoop hardness

Baseline Knoop hardness values were measured using a Microhardness Tester HMV-G series (Shimadzu HMV-G31D, Japan) on 30 samples, each mounted in acrylic blocks, applying a 200 g load for 15 seconds. The samples were then submerged in a glass container containing 50 mL of demineralizing solution and kept at room temperature for 72 hours. Following demineralization, a Knoop indenter was employed to assess the presence of subsurface lesions on the tooth surfaces [6].

**Figure 1:**
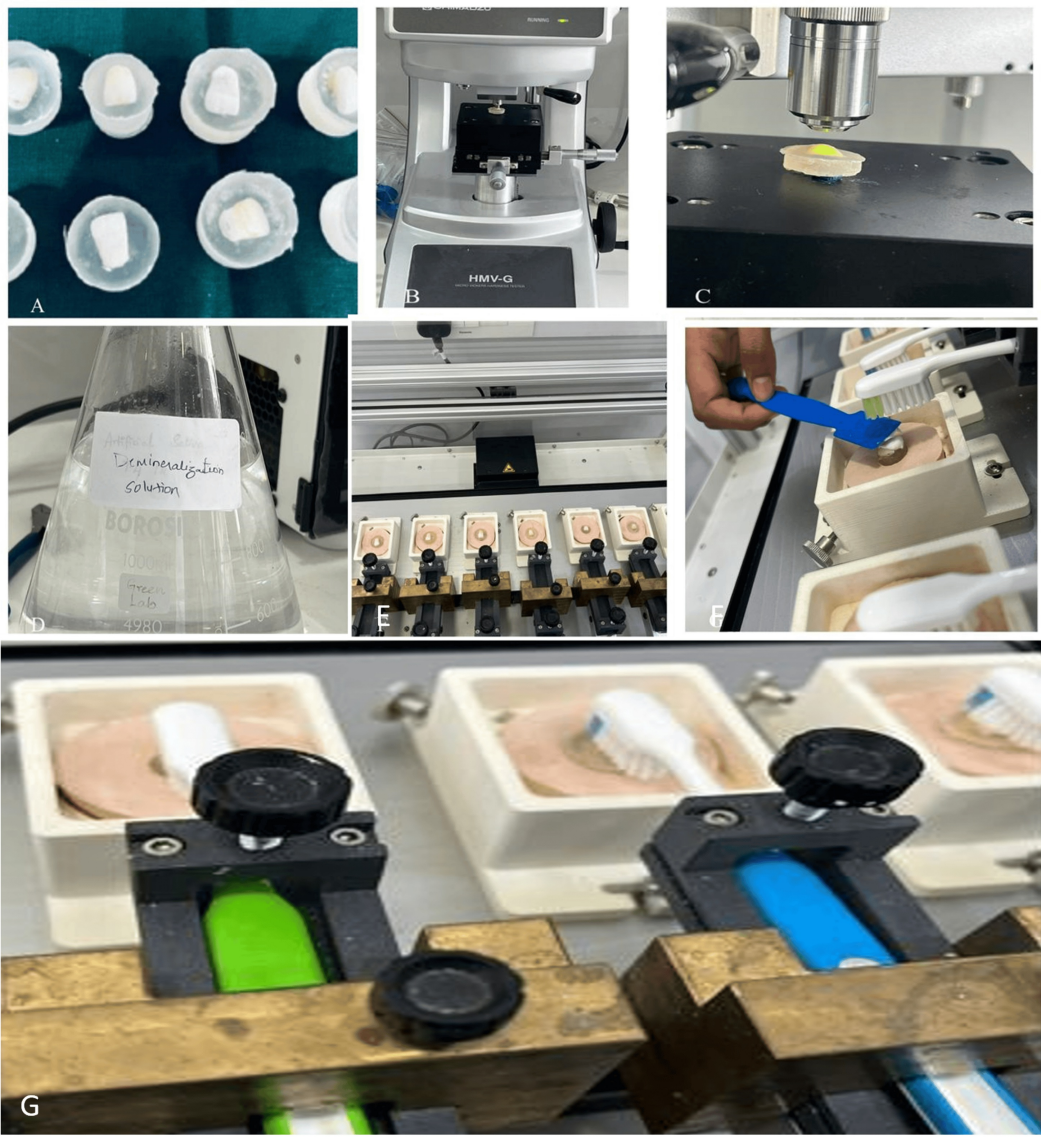
The diagrammatic representation of the methodology used: (A) samples; (B and C) checking the KHN; (D) preparation of demineralization solution; (E) samples placed on the simulator; (F) toothpastes applied over the samples; and (G) brushing by toothbrush simulator KHN: Knoop hardness number

The 30 samples were divided into three groups for the remineralization phase. Group 1 served as the Control, Group 2 was treated with EX remineralizing toothpaste, and Group 3 with Sensodyne remineralizing toothpaste. A brushing simulator (ZM-3.8, Germany) was used to simulate a year-long brushing cycle, consisting of 4000 linear strokes in the X direction, 4000 linear strokes in the Y direction, 1000 clockwise rotations, and 1000 counterclockwise rotations. After the remineralization treatment, the samples were again mounted in acrylic blocks, and the changes in enamel hardness were assessed using a Knoop indenter. The electropolishing was done before and after treatment with demineralizing solution and remineralization toothpaste. A diagrammatic representation of the entire procedure is provided in Figure 1.

## Results

The study evaluated the efficacy of two remineralizing toothpastes, EX and SR, in restoring enamel microhardness. Enamel microhardness was measured using the KHN to assess the results.

Microhardness measurements

Baseline Knoop Hardness Number

The initial KHN values for the enamel samples were recorded to establish a baseline for comparison. All samples demonstrated consistent microhardness levels before demineralization.

Post-Demineralization Knoop Hardness Number

Following the demineralization process, a significant reduction in KHN was observed across all samples, indicating the loss of mineral content and subsequent weakening of the enamel structure.

Post-Remineralization Knoop Hardness Number

Enafix: After the application of EX, the KHN values showed a marked increase, indicating effective remineralization. The microhardness of the enamel treated with EX approached near-baseline levels when compared to the Control group, reflecting its capacity to restore enamel strength.

Sensodyne Repair: Similarly, the application of SR resulted in a substantial increase in KHN, indicating successful remineralization. The microhardness of the enamel treated with SR also approached near-baseline levels.

Comparative analysis and statistical analysis

The comparison between the two remineralizing agents revealed that both EX and SR were effective in enhancing the enamel microhardness after demineralization. In the analysis, the Control group exhibited the highest mean score of 472.00 with a relatively low standard deviation of 17.783, indicating lower variability compared to the other groups. In contrast, the EX group demonstrated a lower mean score of 340.40 and higher variability with a standard deviation of 40.368. The SR group showed the lowest mean score of 260.40 with a moderate level of variability, as indicated by a standard deviation of 34.076. Considering all groups combined, the overall mean was 357.60 with a standard deviation of 94.005, reflecting a wider spread of values and highlighting significant differences between the groups. The 95% confidence intervals for each group did not overlap significantly, further supporting the observed differences in the post-hoc analysis (Table 1).

**Table 1:** The mean and standard deviation among the three groups: Control, EX, and SR Ex: Enafix; SR: Sensodyne Repair

Samples	N	Mean	Std. deviation	Std. error	95% confidence interval for mean		Minimum	Maximum
					Lower bound	Upper bound		
Control	10	472.00	17.783	5.623	459.28	484.72	445	498
EX	10	340.40	40.368	12.766	311.52	369.28	289	421
SR	10	260.40	34.076	10.776	236.02	284.78	221	324
Total	30	357.60	94.005	17.163	322.50	392.70	221	498

**Figure 2:**
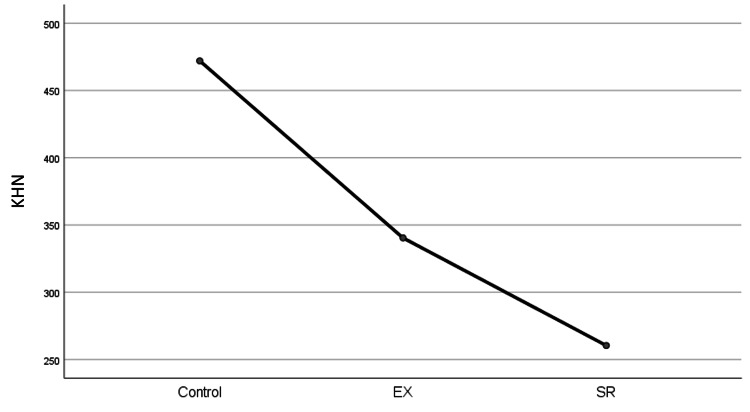
Graph represents the KHN of the samples after remineralization with the toothpaste: EX-340.40, SR-260.40, and Control -472. The X-axis represents the sample groups, and the Y-axis represents KHN KHN: Knoop hardness number; EX: Enafix; SR: Sensodyne Repair

The graph shows a descending trend, where the mean values decrease progressively from the Control group to the SR group (Figure 2).

The data followed a normal distribution, confirming its parametric nature, and we applied a t-test for the analysis. In all pairwise comparisons, statistical significance is evident, with p-values of 0.000 ( which is less than 0.005), falling below the 0.05 threshold (Table 2). This implies notable differences in the dependent variable among all group pairs (Control versus. EX, Control versus SR, and EX versus SR). The Control group exhibits the highest mean, succeeded by the EX group, and the SR group displays the lowest mean. The observed differences in means across these groups are sufficiently large to be deemed statistically significant, denoted by non-overlapping confidence intervals and low p-values.

**Table 2:** Statistical analysis using a t-test (parametric) showing differences among the three groups with the highest mean in the Control and the lowest in the SR group ^*^The mean difference is significant at the 0.05 level. EX: Enafix; SR: Sensodyne Repair

Samples	Comparison	Mean difference (I-J)	Std. error	Sig.	95% confidence interval	
					Lower bound	Upper bound
Control	EX	131.600^*^	14.392	.000	95.92	167.28
	SR	211.600^*^	14.392	.000	175.92	247.28
EX	Control	-131.600^*^	14.392	.000	-167.28	-95.92
	SR	80.000^*^	14.392	.000	44.32	115.68
SR	Control	-211.600^*^	14.392	.000	-247.28	-175.92
	EX	-80.000^*^	14.392	.000	-115.68	-44.32

The analysis of variance (ANOVA) findings reveals a statistically significant distinction in the dependent variable among the three groups (Control, EX, and SR). The F-statistic is 104.292, accompanied by a p-value of 0.000, indicating highly significant differences in the group means (Table 3). This signifies a substantial impact of group membership on the dependent variable.

**Table 3:** The significant difference between the groups using ANOVA and it is statistically significant Df: degree of freedom; ANOVA: analysis of variance

Comparison	Sum of squares	Df	Mean square	F	Sig.
Between Groups	220016.012	2	110008.006	104.292	.000
Within Groups	27425.022	27	1054.809		

## Discussion

The study aimed to evaluate and compare the efficacy of two remineralizing toothpastes, EX and SR, in restoring enamel microhardness, as measured by the KHN. The ability of both EX and SR to significantly enhance enamel microhardness post-demineralization highlights their effectiveness in promoting remineralization. The findings indicated that both remineralizing agents effectively increased the enamel microhardness following demineralization, with EX showing a marginally higher, yet not statistically significant, microhardness compared to SR. This discussion delves into the implications of these findings, comparing them with similar studies that have utilized toothbrush simulators to assess the efficacy of remineralizing toothpaste.

Previous studies have employed toothbrush simulators to assess the efficacy of various remineralizing toothpastes, offering a standardized and reproducible method to simulate the mechanical action of toothbrushing combined with the chemical effects of the toothpaste [9]. Research indicates that toothpastes containing 8% arginine and calcium carbonate, such as Colgate Sensitive Pro-Relief, demonstrate superior remineralization capabilities, achieving a KHN recovery of approximately 44.53% after treatment, compared to lower recoveries from other products like Crest Cavity Protection (24.56%) and GC Tooth Mousse (35.00%) [10]. Similarly, another study found that Colgate Sensitive Pro-Relief and NovaMin® toothpaste significantly enhanced surface hardness compared to GC Tooth Mousse [11]. Additionally, remineralizing agents like casein phosphopeptide-amorphous calcium phosphate (CPP-ACP) also show promising results, although they may not outperform the aforementioned formulations [12]. Also, the active ingredient of EX was Sodium mono fluorophosphate and SR was Calcium Sodium Phosphosilicate with effective remineralizing capacity. Overall, the effectiveness of these toothpastes in enhancing enamel hardness underscores their potential in caries prevention and enamel restoration.

Toothpaste containing fluoride has been shown to significantly increase enamel microhardness, a finding consistent with the results of this study [13]. This supports the importance of fluoride in promoting remineralization, as both toothpastes used in this study include fluoride. Additionally, research indicates that toothpastes fortified with calcium and phosphate-based technologies, alongside fluoride, offer greater improvement in enamel remineralization compared to fluoride alone. This finding corroborates the current study's results, where both EX and SR, which incorporate additional remineralizing agents like calcium phosphates and NovaMin, effectively restored enamel microhardness [14,15]. The marginally greater efficacy observed with EX may be due to variations in the composition of these supplementary agents.

The study assessed the effectiveness of toothpaste containing bioactive glass (NovaMin) in remineralizing enamel using a toothbrush simulator. Results demonstrated significant improvements in enamel microhardness, aligning with previous findings for SR, which also contains NovaMin. This comparison reinforces the efficacy of NovaMin-based formulations in enamel remineralization, supporting SR’s performance in the present study [16].

Additionally, a prior study evaluating toothpaste containing nano-hydroxyapatite using a toothbrush simulator showed that nano-hydroxyapatite is highly effective in remineralizing enamel and enhancing microhardness [17]. Although both toothpastes do not contain nano-hydroxyapatite, the comparison is relevant as it highlights the potential of innovative remineralizing agents to deliver similar or superior results. The slight difference observed in the toothpaste containing sodium mono fluorophosphate in this study highlights its potential [18].

Findings from this study suggest that both toothpastes are effective for remineralizing demineralized enamel, offering comparable outcomes in restoring enamel microhardness. Toothpaste containing sodium mono fluorophosphate's slight advantage may be clinically significant in situations where enhanced remineralization is critical, such as in patients with high caries risk. However, the clinical relevance of the minor difference between the two toothpastes warrants careful consideration. Both formulations are likely to be beneficial in remineralizing early carious lesions and preventing further enamel demineralization when incorporated into a comprehensive oral hygiene routine.

Limitations of the study

While this study provides important insights into the efficacy of these remineralizing agents, several limitations must be noted. Conducted in vitro, the findings may not fully replicate the complexities of the oral environment, where factors such as saliva flow, pH changes, and dietary habits influence the remineralization process. Additionally, although a toothbrush simulator is a standardized method, it may not accurately reflect variations in brushing technique and pressure experienced in everyday use. Future research should prioritize clinical trials to confirm these results in vivo, with a focus on long-term outcomes and patient-centered factors like compliance and sensitivity. Exploring the potential of emerging remineralizing technologies, such as nano-hydroxyapatite and peptide-based formulations, could further enhance preventive dental care.

## Conclusions

The study shows that both remineralizing agents effectively enhance enamel microhardness following demineralization, with one exhibiting a marginally greater effect due to its higher fluoride content of 1450 ppm in sodium mono fluorophosphate compared to the other’s 1000 ppm in calcium sodium phosphosilicate. These results suggest that both products are effective in supporting enamel remineralization. However, further in vivo research is required to evaluate their real-world efficacy, taking into account variables like salivary flow and diet. Despite this, both agents hold promise for strengthening enamel and preventing tooth decay.
